# Clot time ratio (CTR) and relation to treatment outcome in patients with atrial fibrillation treated with Rivaroxaban

**DOI:** 10.1186/s12959-024-00591-x

**Published:** 2024-03-01

**Authors:** Liselotte Onelöv, Elvar Theodorsson, Mojca Božič-Mijovski†, Alenka Mavri

**Affiliations:** 1 Nordic Biomarker, Linköping, Sweden; 2 Department of Vascular Diseases, University Medical Centre Ljubljana, Ljubljana, Slovenia

**Keywords:** Rivaroxaban, Atrial fibrillation, PT DOAC, Clinical outcome, Bleeds, Thrombosis, Laboratory practice

## Abstract

**Background:**

There are situations where information about the anticoagulant effects of Rivaroxaban could be clinically useful. Methods for measuring Rivaroxaban concentrations are not available at all medical laboratories while the test MRX PT DOAC for measuring the functional effects of Rivaroxaban, in CTR (Clot Time Ratio), can be made available around the clock. The objectives of this study were to investigate CTR in trough and peak samples during Rivaroxaban treatment of atrial fibrillation and to correlate the findings to bleeding episodes.

**Methods:**

3 trough- and 3 peak samples from 60 patients (30 on 20 mg daily and 30 on 15 mg daily) were analyzed with PT DOAC. Patients were monitored for 20 months, and bleeding and thrombotic events were documented. Descriptive statistics were used to summarize the data and non-parametric t-test for comparison between groups. ROC curves for the prediction of DOAC plasma levels > 50 ng/mL as determined with LC-MS/MS and anti-FXa methods were computed.

**Results:**

There was a significant difference between trough and peak CTR (median CTR 1.33 vs. 3.57, *p* < 0.001). 28 patients suffered bleeds. Patients on 20 mg Rivaroxaban with bleeds had higher mean peak CTR than patients without bleeds (CTR 4.11 vs. CTR 3.47, *p* = 0.040). There was no significant difference in mean CTR between patients on 15 mg Rivaroxaban with or without bleeds (CTR 3.81 vs. 3.21, *p* = 0.803), or when considering all patients (CTR 3.63 vs. 3.56, *p* = 0.445). Five out of seven patients on Rivaroxaban 20 with mean peak CTR above the dose specific first to third quartile range (Q1-Q3) suffered bleeds, while 7/16 patients with mean peak CTR within, and 1/7 patients with mean peak CTR below the Q1-Q3 suffered bleeds. The area under the ROC curve was > 0.98 at the upper limit of the PT DOAC reference interval and the negative predictive value of PT DOAC for the prediction of DOAC plasma levels > 50 ng/mL was > 0.96.

**Conclusions:**

The sample size was too low to draw any firm conclusions but is seems that MRX PT DOAC might be a useful laboratory test in situations where the effect of Rivaroxaban needs evaluation.

## Background

Rivaroxaban (Xarelto®) is used to prevent stroke and systemic embolism in patients with atrial fibrillation (AF) and to treat venous thromboembolism (VTE). Two doses are available for treatment of atrial fibrillation, 20 mg Rivaroxaban once daily, and 15 mg Rivaroxaban once daily for patients with renal insufficiency (creatinine clearance 15–29 ml) [1]. The European Medicines Agency (EMA) states that there is no routine need to monitor the exposure of the drug but that there are clinical situations, such as overdose and emergency surgery, in which information on Rivaroxaban exposure might help in clinical decisions. Standard clotting assays are affected by Rivaroxaban, and different reagents would provide different results. APTT is, however, not recommended for assessing the pharmacodynamic effect of Rivaroxaban by EMA, while prothrombin time can be used if the results are reported in seconds since the INR conversion is calibrated and validated for vitamin K antagonists only. Calibrated chromogenic substrate-based quantitative anti-factor Xa assays can be used for measurement of the Rivaroxaban levels when needed. Although chromogenic anti-FXa assays are commonly available at large hospital laboratories, numerous middle-sized and small laboratories still do not have access to these assays. As the prescription of Direct Oral Anticoagulants (DOACs) is increasing, there is also an increased need for a simple and accurate laboratory test for measuring the effect of DOACs. The test should be available around the clock also at middle-sized and small clinical laboratories to assess the anticoagulant effect of DOACs in emergency situations. According to the 2021 update of the International Council for Standardization of Haematology recommendations for laboratory measurements of Direct Oral Anticoagulant results for urgently ordered DOAC measurements should be available within 30 min to aid in clinical decision-making [2].

The novel assay PT DOAC measures the functional effects of DOACs, expressed in Clot Time Ratio (CTR), and can easily be made available around the clock [3, 4] at any coagulation laboratory. To test the usefulness of PT DOAC in a clinical setting, we investigated the peak and trough CTR in patients treated with Rivaroxaban for atrial fibrillation. Results were correlated to the drug concentration in the samples and, more importantly, to the clinical outcome for the patients.

## Materials and methods

### Patients

Sixty patients treated with Rivaroxaban for atrial fibrillation at the Anticoagulation Clinic (University Medical Centre, Ljubljana, Slovenia) were included in the study. Thirty patients received the full dose of 20 mg of Rivaroxaban once daily (hereafter called ‘Rivaroxaban 20’), and 30 patients received the lower dose of 15 mg of Rivaroxaban once daily (hereafter called ‘Rivaroxaban 15’). The lower dose rivaroxaban was prescribed to patients with moderate renal impairment (CrCl 30–50 mL/min), high bleeding risk or previous major bleeding, at the discretion of the treating physician.

None of the patients received strong P-gp/CYP3A4 inhibitors or inducers.

Patients were monitored for 20 months. Clinical parameters such as age, gender, body weight, creatinine, arterial hypertension, diabetes mellitus, heart failure, ischemic heart disease, previous stroke or systemic embolism, peripheral artery disease, CHADS_2_ score, HAS-BLED score, treatment schedule (Rivaroxaban 20 vs. Rivaroxaban 15) and clinical outcomes such as bleeding events and thromboembolic events during study follow up were recorded. In addition, laboratory data on Rivaroxaban concentration, determined with LC-MS/MS and chromogenic anti-FXa methods, were collected.

The Medical Ethical Committee of the Slovenian Ministry of Health approved the study.

### Sampling

Three trough and three peak samples were collected from each patient at three samplings 6–8 weeks apart. Trough samples were collected 24 +/- 1 h after the previous Rivaroxaban dose, while peak samples were collected 124 +/- 8 min after the previous dose. One patient on the lower dose only attended the first two samplings, while for one patient on the full dose, no excess samples were available for additional laboratory investigation within this study from the first sampling (trough 1 and peak 1). Finally, one sample from a patient on the lower dose was lost during investigation.

### Laboratory parameters

MRX PT DOAC (Nordic Biomarker, Umeå, Sweden) was used to determine CTR in 355 samples on the CS2100i coagulation analyzer (Sysmex, Kobe, Japan), according to the instructions from the manufacturer. Samples were thawed in 37◦C water bath for a few minutes before analysis as they had been stored in -70◦C since collection. At the time of analysis, the laboratory technician did not know the origin of the samples (trough or peak, from a patient with/without bleeds).

LC-MS/MS and anti-FXa analyses of the same samples were performed in an earlier study [5] and the results were correlated to CTR in this study.

### Statistical methods

Categorical variables are presented as counts and percentages, while continuous variables are presented as mean or median with range min-max or first to third quartile (Q1-Q3). The within-patient trough and peak coefficient of variation (CV) were calculated as standard deviation/average x 100 from all available trough- and peak measurements of each patient. A statistical comparison was performed by using a t-test or Mann-Whitney Rank Sum test for the continuous variables depending on if the assumption of normality was fulfilled or not, and for the categorical variables with a chi^2^ test or depending on sample size a Fisher exact test. A Pearson Correlation test was used to evaluate correlations between laboratory result and patient characteristics. Sensitivity, specificity, positive- and negative predictive value for PT DOAC CTR at the upper limit of the reference range (CTR 1.38, provided by the manufacturer) to predict DOAC levels > 50 ng/mL, as determined with LC-MS/MS and anti-FXa were calculated. Receiver operator curves (ROC) were computed and the area under the curve calculated with 95% confidence interval. Statistical evaluations of results were done with SigmaPlot 14.0 and MS Excel. Two-sided *p* < 0.05 was considered statistically significant.

## Results

### Patient demographics

The two patient groups (Rivaroxaban 20 and Rivaroxaban 15) were compared according to demographics. Patients in the group with the reduced Rivaroxaban dose were older, weighed less, and had higher CHA_2_DS_2_VASc and HAS-BLED scores than patients in the full dose group (Table 1). In addition, creatinine (µmol/L) and creatinine clearance (CrCl) (mL/min), were previously reported to differ between the dose groups [5].

**Table 1:** Characteristics of the patients on Rivaroxaban 20 or Rivaroxaban 15

	Either dose *N* = 60	Rivaroxaban 20 *N* = 30	Rivaroxaban 15 *N* = 30	Rivaroxaban 20 vs. Rivaroxaban 15 *p*-value
Age (years)	73 (59–87)	71 (59–80)	76 (63–87)	< 0.001
Gender, female/male	28/32	9/21	19/11	*p* = 0.02
Weight (kg)	84 (60–140)	90 (60–140)	79 (60–134)	*p* = 0.004
Hypertension	54 (90%)	26 (87%)	28 (93%)	*p* = 0.671
Diabetes	13 (22%)	4 (13%)	9 (30%)	*p* = 0.210
Chronic heart failure	14 (23%)	6 (20%)	8 (27%)	*p* = 0.760
Peripheral Artery Obstructive Disease	2 (3%)	1 (3%)	1 (3%)	*p* = 1.000
Ischemic Heart Disease	12 (20%)	6 (20%)	6 (20%)	*p* = 0.747
Cerebrovascular Insult	8 (13%)	3 (10%)	5 (17%)	*p* = 0.480
CHA_2_DS_2_VASc score:	2.1 +/-1.3	1.8 +/- 1.3	2.5 +/- 1.2	*p* = 0.048
HAS-BLED Score	1.1+/-0.6	0.9 +/-0.7	1.2 +/-0.5	*p* = 0.028
Bleeding events (yes/no)	28	13	15	*p* = 0.796
Bleeding severity (major/minor)	3/25	2/11	1/14	*p* = 0.664
Thromboembolic events	3	2	1	*p* = 1.000

Values presented as mean (range), count (%) or mean +/-sd.

There were no statistically significant differences between patients with and without bleeds regarding age, gender, weight, hypertension, diabetes, chronic heart failure, peripheral artery obstructive disease, ischemic heart disease, cerebrovascular disease, cerebrovascular insult, CHA_2_DS_2_VASc score or HAS-BLED score (Table 2). This was true for patient both on Rivaroxaban 20 and 15.

**Table 2:** Characteristics of the patients with vs. without bleeds

	Rivaroxaban 20			Rivaroxaban 15		
	Without bleeding *N* = 17	With bleeding *N* = 13	*p*-value	Without bleeding *N* = 15	With bleeding *N* = 15	*p*-value
Age (years)	69 (59–78)	73 (61–80)	0.516	77 (64–87)	76 (63–83)	0.130
Gender, female/male	3/14	6/7	0.123	10/5	9/6	1.000
Body weight (kg)	90 (60–112)	89 (64–140)	0.064	73 (60–102)	84 (66–134)	0.909
Hypertension, N (%)	15 (88)	11 (85)	1.000	15 (100)	13 (87)	0.483
Diabetes, N (%)	1 (6)	3 (23)	0.290	5 (33)	4 (27)	1.000
Chronic heart failure, N (%)	4 (24)	2 (15)	0.672	5 (33)	3 (20)	0.682
Peripheral Artery Obstructive Disease, N (%)	1 (6)	0	1.000	1 (7)	0	1.000
Ischemic Heart Disease, N (%)	4 (24)	2 (15)	0.672	3 (20)	3 (20)	1.000
Cerebrovascular Insult, N (%)	2 (12)	1 (8)	1.000	2 (13)	3 (20)	1.000
CHA_2_DS_2_VASc score	1.6 +/-1.2	2.0 +/-1.4	0.668	2.7 +/-1.2	2.3 +/-1.1	0.845
HAS-BLED Score	0.8 +/-0.7	1.1 +/-0.6	0.096	1.3 +/-0.5	1.2 +/-0.6	0.592
No. of bleeding events						
1 2 3 4	0 0 0 0	9 2 1 1		0 0 0 0	6 6 1 2	

Values presented as mean (range), count (%) or mean +/-sd. P-value is for comparison between patients with/without bleeds.

PT DOAC (CTR) was determined in 178 trough and 177 peak samples. Trough CTR was 0.93–3.36 with the median at 1.33, while peak CTR was 1.80–7.03 with the median at 3.57. The CTR was significantly higher in peak samples as compared to trough samples (*p* < 0.001), indicating that the effect of rivaroxaban can be measured using PT DOAC.

**Fig. 1:**
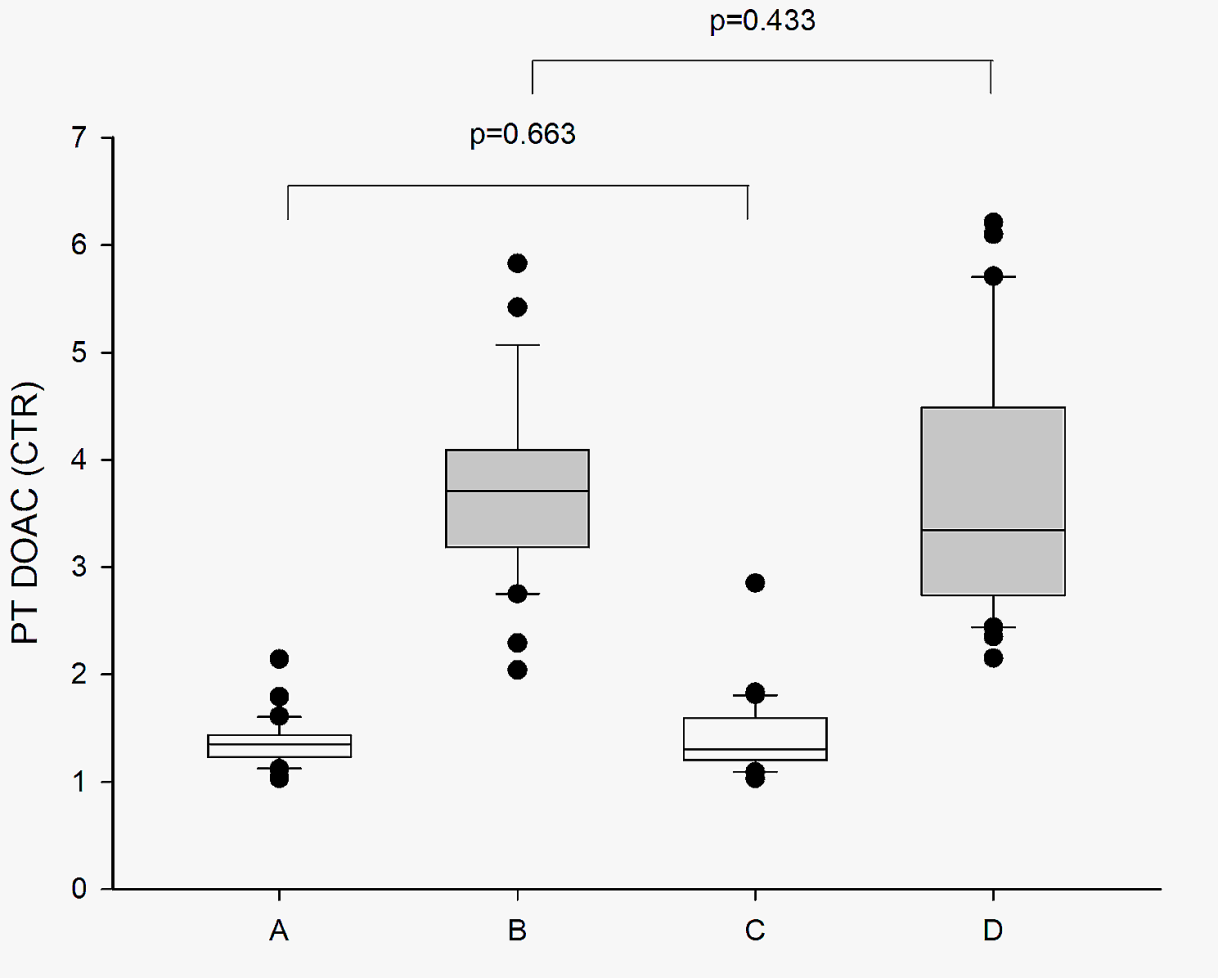
Clot time ratio in trough and peak samples. Comparison of mean CTR at trough (**A, C**) and peak (**B, D**) measured in plasma samples from 30 patients on Rivaroxaban 20 (**A-B**) and 30 patients on Rivaroxaban 15 (**C-D**) using PT DOAC. The bottom and top of the box represent the first and third quartiles (Q1-Q3), while the band inside the box represents the median

Neither trough nor peak CTR differed between patients on Rivaroxaban 20 vs. patients on Rivaroxaban 15 *p* = 0.663 and *p* = 0.443 respectively (Fig. 1), indicating that the effect of rivaroxaban was similar in both patient groups at the time right before the next dose (trough) and that both doses give the same effect in the patients (peak).

We used the first to third quartile range (Q1-Q3) of CTR to evaluate the association with clinical outcomes. No overlap was seen between the trough CTR Q1-Q3 range (T_Q1−Q3_) and the peak Q1-Q3 (P_Q1−Q3_) for either dose-group (Fig. 1). The P_Q1−Q3_ for the lower dose was however wider than for the full dose (CTR 2.7–4.6 and CTR 3.2–4.2 respectively), indicating a larger variation in the effect obtained from the adjusted dose compared to the full dose of Rivaroxaban.

CTR at repeated samplings (through 1, 2, 3, and peak 1, 2, 3) did not vary for patients on Rivaroxaban 15, or at peak for Rivaroxaban 20 (Table 3), while there was a significant difference for CTR at trough for Rivaroxaban 20 at sampling 1 compared to sampling 2 (Table 3). A post-hoc Bonferroni t-test revealed the difference between trough sampling 1 and 2. Further analysis of the results at trough sampling 1 disclosed that one patient (#20) had a very high CTR at trough sampling 1 versus trough sampling 2 and 3. Accordingly, this patient had a higher rivaroxaban concentration at the first sampling than the following samplings (285 ng/mL vs. 35 and 57). Exclusion of this sample from the analysis did however not remove the difference between trough sampling 1 and 2.

Intra-patient CV at trough and peak did not differ between the dose groups (Table 3). Combined CV (both doses) for CTR at trough was lower than CV for CTR at peak (*p* < 0.001).

**Table 3:** Comparison of the effect of rivaroxaban (CTR) measured in 3 trough and 3 peak samples

	Trough 1	Trough 2	Trough 3	ANOVA P	Average CV (%)	Peak 1	Peak 2	Peak 3	ANOVA P	Average CV (%)
**Rivaroxaban 20**										
N Median Min-max	29 1.34 1.08–3.36	30 1.32 0.94–1.81	30 1.34 1.01–1.67	0.029*	30 4.8 0.72–40.7	29 3.77 1.96–6.2	30 3.71 2.10–5.88	30 3.69 1.89–5.49	0.297	30 9.4 1.89–24.9
**Rivaroxaban 15**										
N Median Min-max	30 1.32 1.02–2.53	30 1.30 1.03–2.78	29 1.37 0.93–3.23	0.914	30 4.61 1.61–24.7	30 3.53 2.08–6.58	30 3.27 1.89–6.17	28 3.14 1.80–7.03	0.303	30 8.34 0.56–28.1

Number of samples (N), median and range (min-max) are presented for each sampling, and the average coefficient of variation (CV) between them are shown. Result from repeated measure analysis is presented with ANOVA P.

CTR at peak did not correlate with age or body weight, and while mean peak CTR was lower in patients with cerebrovascular disease (*p* = 0.025) compared to patients without, no differences were seen between patients with/without any of the following patient characteristics: hypertension, diabetes, chronic heart failure, peripheral artery obstructive disease, ischemic heart disease, CHA_2_DS_2_VASC score, HAS-BLED, bleedings or thrombotic events. Mean trough CTR did not correlate to any of the evaluated parameters.

**Fig. 2:**
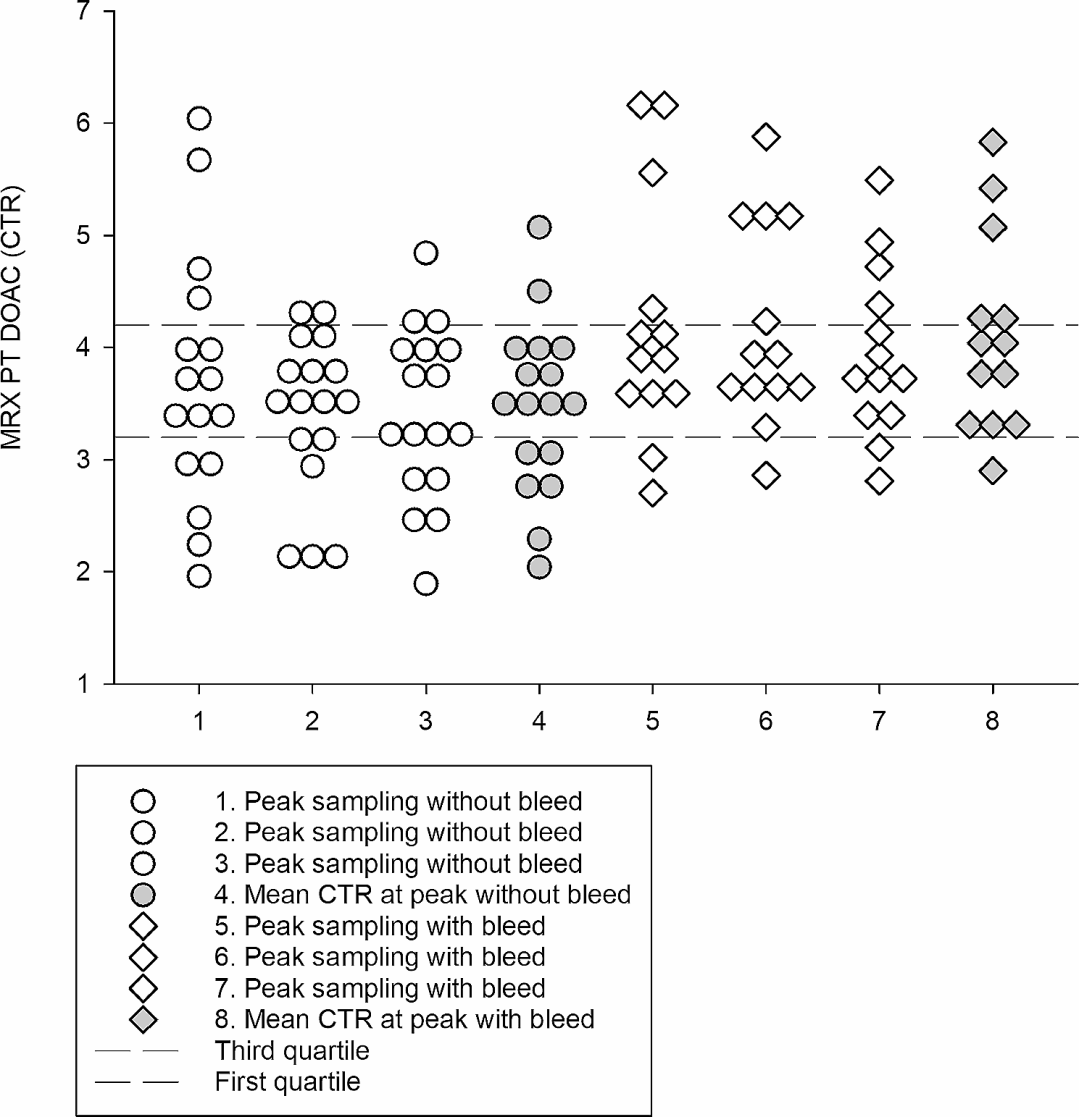
Clot time ratio in patients without and with bleeds. Individual CTRs (white) from sampling 1, 2 and 3 and the mean CTR (grey) for patients on Rivaroxaban 20 without bleeds (circles) and with bleeds (diamonds). The dashed lines represent the first and third quartiles (P_Q1−Q3_) for patients on 20 mg Rivaroxaban

During the study, 28 patients (13 on 20 mg and 15 on 15 mg rivaroxaban) suffered bleeding. Three patients suffered major bleedings, and 25 suffered 1–4 minor ones [5]. There was no difference in mean peak- or trough CTR between patients with- and without bleeds, *p* = 0.445 and *p* = 0.202 respectively. When looking at the results for the two dose groups separately it was found that patients on Rivaroxaban 20 with bleeds had higher mean peak CTR than patients without bleeds (*p* = 0.040). In comparison, there was no significant difference in mean peak CTR between patients on Rivaroxaban 15 with or without bleeds (*p* = 0.803) or at trough for any of the dose groups (*p* = 0.209, *p* = 0.648, Fig. 2).

Five out of seven patients (71%) on Rivaroxaban 20 with mean peak CTR above the dose specific P_Q1−Q3_ suffered bleeds while 7/16 patients (44%) with mean peak CTR within, and 1/7 patients (14%) with mean peak CTR below the P_Q1−Q3_ suffered bleeds indicating a higher risk of suffering bleeds on full dose rivaroxaban reflected in higher CTR at peak. In addition, among the rivaroxaban 20 non-bleeders only 1 had peak CTRs that were consistently above the P_Q1−Q3_ while the remaining 5 patients had a single peak CTR above the P_Q1−Q3_. On the other hand, among the Rivaroxaban 20 bleeders, 4 patients had peak CTRs consistently above the P_Q1−Q3_, one patient had 2/3 peak CTRs above the P_Q1−Q3_, while 2 patients had a single CTR above P_Q1−Q3_. Figure 2 shows the distribution of peak CTRs for individual samples at sampling 1, 2 and 3 as well as the patient mean CTRs for patients on Rivaroxaban 20 without and with bleeds.

For patients on Rivaroxaban 15, 3/7 patients (43%) with mean peak CTR above the dose specific P_Q1−Q3_, 8/16 (50%) with mean peak CTR within the P_Q1−Q3_ and 4/7 patients (57%) with mean peak CTR below the P_Q1−Q3_ suffered bleeds. The risk of suffering bleeds was similar regardless of CTR for this patient group.

Three patients suffered thrombotic events during the study (two on Rivaroxaban 20 and one on Rivaroxaban 15). One of the patients on Rivaroxaban 20 also suffered a minor bleed. As only three patients were suffering from thrombotic events, and their mean CTR at trough and peak varied with the respective Q1-Q3, no conclusions can be drawn regarding CTR and thrombotic events.

**Fig. 3:**
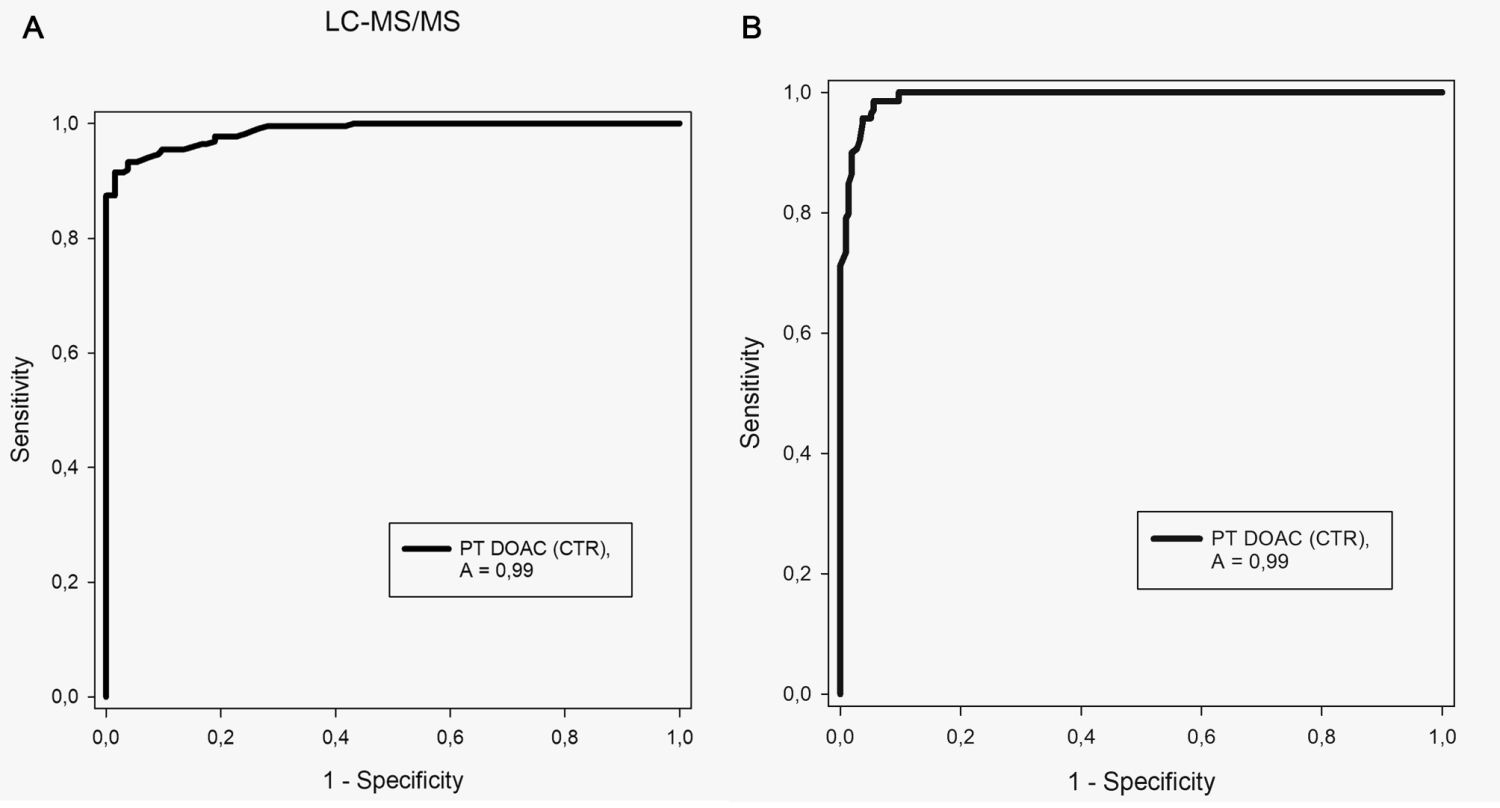
ROC curves. ROC curves for the prediction of Rivaroxaban concentration > 50 ng/mL A; LC-MS/MS and B; Anti-FXa

Rivaroxaban plasma levels measured by LC-MS/MS (ng/mL) or anti-FXa (ng/mL) were available for 355 samples. There was a significant correlation between CTR, and the rivaroxaban plasma levels (*r* = 0.905, *p* < 0.001 for LC-MS/MS and *r* = 0.892, *p* < 0.001 for anti-FXa. The sensitivity, specificity, positive- and negative predictive values, and the area under the curve of the ROC for PT DOAC CTR at the upper limit of the reference interval to predict a rivaroxaban concentration > 50 ng/mL are shown in Table 4. The resulting ROC curves for the prediction of DOAC plasma levels > 50 ng/mL as determined with LC-MS/MS and anti-FXa methods are shown in Fig. 3.

**Table 4:** Sensitivity, specificity, positive- and negative predictive value and area under the curve for prediction of Rivaroxaban plasma concentration > 50 ng/mL

	*N* > 50 ng/mL	Sensitivity (95% CI)	Specificity (95% CI)	PPV	NPV	AUC (95% CI)
**LC-MS/MS**	223	0.9776 (0.9485–0.9927)	0.7727 (0.6917–0.8411)	0.3912	0.9957	0.9866 (0.9785–0.9947)
**Anti-FXa**	216	0.7554 (0.6753–0.8243)	0.9907 (0.9670–0.9989)	0.9239	0.9644	0.9929 (0.9876–0.9982)

Number of samples (N), Positive predictive value (PPT), Negative predictive value (NPV), Area under the Curve (AUC).

## Discussion

The effect of rivaroxaban treatment in patients with AF, measured using PT DOAC, was investigated, and related to relevant patient characteristics, other laboratory parameters, as well as clinical outcomes. The effect of rivaroxaban can be monitored using PT DOAC as the CTR is significantly higher at peak compared to at trough, with no overlap in first to third quartile ranges (Q1-Q3) between trough and peak measurements. Further, in this study, there was no difference between patients on Rivaroxaban 20 vs. 15 at trough or peak CTR. Thus, patients gain the same effect of rivaroxaban as expressed in CTR, regardless of dose. However, the Q1-Q3 was wider for patients on 15, indicating a larger variation in effect between patients on the reduced dose.

Intra-patient coefficient of variation for CTR was higher at peak than at trough. While the relevance for this requires further investigation, this may be due to the pharmacodynamics at the patient level during the first hours after drug intake (samplings were performed 2 h post drug intake) [6], or due to the extrapolation of the calibration curve above CTR 4. Higher CV at peak than at trough was also reported previously for the intra-patient rivaroxaban concentration coefficient of variation in the same patients [5].

Twenty-eight patients suffered 1–4 bleedings during the study, three of them suffered major bleeds, and 25 minor bleeds. For the patients on Rivaroxaban 20, a higher mean CTR at peak indicated a significantly higher risk of suffering bleeds. This was not observed for patients on the reduced dose Rivaroxaban 15 or when combining the two dose groups. Also true for the patients on Rivaroxaban 20 was the percentage-wise higher proportion of bleedings in patients with mean CTR above the dose specific Q1-Q3 as compared to patients with mean CTR within or below the Q1-Q3. The pattern with more bleeds among patients with peak CTRs consistently above the P_Q1−Q3_ might be interpreted as an increased risk for these patients as compared to patients with a single high CTR, and an indication for monitoring the effect or rivaroxaban. However, as this was not true for the whole patient group, and as the Q1-Q3 range was much wider for patients on 15 mg/mL this needs to be further investigated before any firm conclusions can be drawn.

CTR correlated to rivaroxaban plasma levels when measured by LC-MS/MS (*r* = 0.905) and with anti-FXa methods (*r* = 0.892), indicating that PT DOAC might be used in situations where these tests are not available. A negative predictive value at the upper limit of PT DOAC reference interval of > 0.96, and area under the ROC curve at > 0.98 further strengthens the usefulness of PT DOAC. Several studies have tried to correlate rivaroxaban concentration to clinical outcomes, as reviewed in a recent letter to the editors [7]. For example, while Jaowenko et al. saw no correlation to the concentration, but identified advanced age, inappropriately high dosing regimens, and modest peak anti-FXa rivaroxaban levels to be associated with major bleeding [8], both Testa et al. and Wada et al. [9–11] on the other hand found that the rivaroxaban concentration was higher in patients with bleedings compared to patients without bleedings.

DOACs exert the expected inhibitory effects on most clotting tests, but these functional effects have yet not found widespread clinical use [12–14]. Tests based on the dilute Russell’s viper venom time (dRVVT) have been shown to be sensitive to DOACs and claimed to be promising for clinical use [15–19]. However, clinical studies of these tests related to the occurrence of bleeding and their relations to drug concentrations are still in exploratory stages [17, 18].

The results of the present clinical study of rivaroxaban and bleedings combined with a comparative study of CTR and rivaroxaban concentrations, represents a further approach in using functional coagulation assays for monitoring the risk of bleeding caused by DOACs. PT DOAC for measuring the functional effects of rivaroxaban uses the principles of the prothrombin time test in a CTR and is available for widely available measuring systems for prothrombin time. While methods for measuring the concentrations of rivaroxaban are primarily available in large laboratories the functional CTR can be performed even in medium sized laboratories using already available measuring systems.

## Conclusion

The area under the ROC curve for the prediction of DOAC plasma levels > 50 ng/mL and the negative predictive value suggests that MRX PT DOAC is a useful laboratory test in situations where the effect of Rivaroxaban needs evaluation. Patients on full dose rivaroxaban with peak CTR above the dose specific P_Q1−Q3_ might have a higher risk of suffering bleeds than patients with peak CTR within or below Q1-Q3.

## Data Availability

The datasets used and/or analyzed during the current study are available from the corresponding author on reasonable request.

## Abbreviations

CTR: Clot Time Ratio
Q1-Q3: First to third quartile range
T_Q1−Q3_: First to third quartile range at trough
P_Q1−Q3_: First to third quartile range at peak
AF: Atrial Fibrillation
VTE: Venous ThromboEmbolism
EMA: European Medicine Agency
DOAC: Direct Oral AntiCoagulants
